# Profiling the Cerebrospinal Fluid Proteome in Progressive Multiple Sclerosis: Treatment Effects and Associations with IgM Oligoclonal Bands

**DOI:** 10.1007/s11481-025-10263-w

**Published:** 2025-10-30

**Authors:** Sahla El Mahdaoui, Peter Kosa, Mika Komori, José Luis Veiga González, Helene Højsgaard Chow, Rikke Ratzer, Camilla Gøbel Madsen, Hartwig Roman Siebner, Bibi Bielekova, Luisa María Villar, Jeppe Romme Christensen, Finn Sellebjerg

**Affiliations:** 1 Danish Multiple Sclerosis Center, Department of Neurology, Copenhagen University Hospital – Rigshospitalet, Glostrup, Denmark; 2 Neuroimmunological Diseases Section, National Institute of Allergy and Infectious Diseases, National Institutes of Health, Bethesda, MD USA; 3 Department of Immunology, Hospital Universitario Ramón y Cajal, IRYCIS, Madrid, Spain; 4 Danish Research Centre for Magnetic Resonance, Department of Radiology and Nuclear Medicine, Copenhagen University Hospital - Amager and Hvidovre, Hvidovre, Denmark; 5 Department of Neurology, Copenhagen University Hospital - Bispebjerg and Frederiksberg, Copenhagen, Denmark; 6 Department of Clinical Medicine, Faculty of Health and Medical Sciences, University of Copenhagen, Copenhagen, Denmark

**Keywords:** Disease-modifying therapy, DMT, Phase 2, Trial, Proteomics, Intervention

## Abstract

Treatment options for progressive MS (PMS) are limited in numbers and efficacy, which is most pronounced in patients with inflammatory disease activity. Immunoglobulin M (IgM) oligoclonal bands (OCBs) may identify a subset of PMS with more active inflammatory disease. The effects of natalizumab and methylprednisolone on intrathecal inflammation and the association of IgM OCBs with other biomarkers in PMS is uncertain. In the current study, we investigated the cerebrospinal fluid (CSF) proteome of untreated patients with PMS, effects of natalizumab and methylprednisolone, and associations of IgM OCBs with disease activity and CSF biomarkers. We found a reduction of BCMA, SLAMF7, granzyme A, IgG, and desmoglein-2 with both therapies, as well as natalizumab-specific reductions of VCAM-1, CD48, MDC, MMP-9, sE-selectin, and CHIT1, and methylprednisolone-specific reductions of DR3, IgD, RTN4, and increases of sCD206, LYVE1, sCD163 and MMP-3. IgM OCBs were associated with reduced levels of PIGR, higher levels of NFL and VEGF, and more contrast-enhancing lesions. The study suggests T and B cell activity biomarkers as treatment-responsive CSF biomarkers in PMS. Additionally, we found natalizumab to reduce adhesion molecules and methylprednisolone to increase myeloid biomarkers. Lastly, we confirm that IgM OCBs are associated with a more inflammatory MRI and CSF profile.

**Graphical Abstract:**

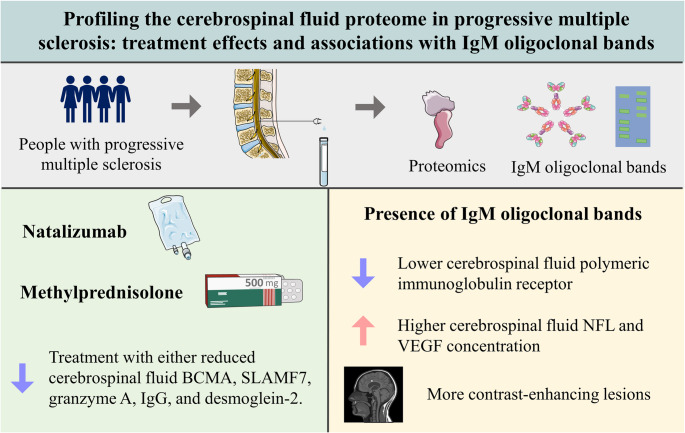

**Supplementary Information:**

The online version contains supplementary material available at 10.1007/s11481-025-10263-w.

## Introduction

Multiple sclerosis (MS) is a chronic disorder of the central nervous system (CNS) causing immune-mediated demyelination and neuroaxonal damage (Reich et al. 2018). MS typically begins as relapsing-remitting (RRMS), characterized by episodic neurological symptoms followed by complete or partial recovery. RRMS may later transition to a progressive phase, termed secondary progressive MS (SPMS). In approximately 10% of cases, MS presents as primary progressive (PPMS) around the same time when people with RRMS convert to SPMS (i.e., 4th to 6th decade), with steady disability progression and accumulation of neurological symptoms from disease onset (Lublin et al. 2014).

Disease-modifying therapies (DMTs) for progressive MS (PMS) have limited efficacy on disability progression, mainly confined to younger patients with evidence of inflammatory disease activity (Kappos et al. 2018; Montalban et al. 2017; Sorensen et al. 2020; Weideman et al. 2017). Cerebrospinal fluid (CSF)-restricted immunoglobulin M (IgM) oligoclonal bands (OCBs) have been proposed as a potential marker for identifying a subset of patients with PPMS with more active inflammatory disease (Villar et al. 2014). While most people with MS have CSF-restricted IgG OCBs, IgM OCBs are less common (Pfuhl et al. 2019; Villar et al. 2003, 2014). Due to their high prevalence, the prognostic value of IgG OCBs is limited. In contrast, the presence of IgM OCBs has been linked to increased relapse risk, shorter relapse intervals, faster disability progression, and conversion from RRMS to SPMS (Villar et al. 2002, 2003, 2005).

CSF examination offers insights into pathophysiological mechanisms in MS and the mode of action of DMTs. We previously conducted two open-label phase 2 A trials investigating the effects of natalizumab and methylprednisolone treatment on selected CSF biomarkers in PMS (Ratzer et al. 2016; Romme Christensen et al. 2014). Natalizumab is a monoclonal antibody directed against the integrin very late antigen-4 (VLA-4), which impairs the migration of immune cells to the CNS (Sellebjerg and Sørensen 2015). Methylprednisolone has a range of effects, including broad immunosuppressive effects and restoration of blood-brain-barrier integrity (Ratzer et al. 2014; Sloka and Stefanelli 2005).

The effects of treatment with natalizumab and methylprednisolone on intrathecal inflammation in PMS are, however, incompletely understood. In addition, the association of IgM OCBs with other biomarkers in PMS is uncertain. Previous studies have used various platforms for proteomic analysis to identify molecules differentially expressed in RRMS and PMS (Åkesson et al. 2023a; Barbour et al. 2017), but CSF studies of treatment effects are sparse and mainly examine selected biomarkers or RRMS (Lereim et al. 2024; Lycke and Zetterberg 2017; Stoop et al. 2013). Motivated by this, we investigated the proteomic profile of untreated patients with SPMS and PPMS, effects of natalizumab and methylprednisolone treatment, and associations with IgM OCBs, identifying treatment-responsive biomarkers in PMS and further characterizing the subsets of patients with IgM OCBs.

## Materials and Methods

### Study Participants and Intervention

In this exploratory study, we investigated patients with PPMS or SPMS who had participated in two open-label phase 2 trials of natalizumab treatment (*n* = 17) or monthly methylprednisolone pulse treatment (*n* = 23) and undergone longitudinal lumbar punctures at baseline and week 60 (Ratzer et al. 2016; Romme Christensen et al. 2014). Natalizumab was given as intravenous infusions of 300 mg every fourth week, and methylprednisolone was administered orally as 500 mg of methylprednisolone daily for three days every fourth week. At baseline, the participants had not received immunomodulatory treatment for at least 3 months or immunosuppressive treatment for at least 6 months. A full description of inclusion and exclusion criteria, study visits and procedures has been published (Ratzer et al. 2016; Romme Christensen et al. 2014). In the natalizumab cohort, 12 were treatment-naïve before inclusion, and the latest DMTs for previously treated were interferon-β (*n* = 3), mitoxantrone (*n* = 1), and methotrexate (*n* = 1). For the methylprednisolone cohort, 8 were treatment-naïve, and the latest DMTs for previously treated were interferon-β (*n* = 7), natalizumab (*n* = 3), mitoxantrone (*n* = 4), and methotrexate (*n* = 1).

Two patients had participated in both trials; the natalizumab trial first followed by the methylprednisolone trial. For pooled characterization of the CSF biomarker profile of patients with and without IgM OCBs, these two participants were only included with their natalizumab trial samples.

### Protocol Approvals, Registrations, and Participant Consent

The studies are registered on clinicaltrials.gov (NCT01077466; 2010-02−26, and NCT01305837; 2011-02−28). Ethical approval was granted for each study by the local ethics committee and the Danish Medicine Agency. The studies were monitored by an independent Good Clinical Practice unit, followed the Declaration of Helsinki, and all participants signed a written informed consent form.

### CSF Sampling

The CSF was collected in a polypropylene tube on ice and immediately centrifuged for 10 min at 400 g (Teunissen et al. 2009). The cell-free CSF supernatant was frozen and stored at −80 °C until proteomic, enzyme-linked immunosorbent assay (ELISA) and electrochemiluminescence assay (ECL) analyses. CSF used for IgM OCB analysis had undergone one or two thaw-freeze cycles.

### Proteomic Analysis

The relative CSF concentration of 1,128 proteins was determined for baseline and week 60 samples using DNA aptamer–based SOMAscan methodology (SomaLogic Inc., Boulder, CO) at the National Institutes of Health (NIH), Bethesda, MD as previously described (Barbour et al. 2017; Gold et al. 2012).

### IgM Oligoclonal Bands

The presence of CSF-restricted IgM OCBs was analyzed for paired serum or plasma and CSF samples using isoelectric focusing and immunoblotting as previously described (Villar et al. 2001).

### Additional Biomarker Analyses

Concentrations of soluble CD27 (sCD27), sCD21, sCD14 and sCD163 were measured in CSF using ECL assays (Meso Scale Discovery platform) at NIH as previously described (Komori et al. 2015; Romme Christensen et al. 2019). CSF neurofilament light chain (NFL), myelin basic protein (MBP) and chitinase 3–like 1 (CHI3L1) concentrations were measured by commercially available ELISA assays from Uman Diagnostics (Umeå, Sweden), Beckman Coulter (Brea, USA) and R&D (Abingdon, UK), respectively. Interleukin-12 subunit p40 (IL12p40) and vascular endothelial growth factor (VEGF) were measured using ECL V-PLEX panels. All concentrations were above the lower limits of detection (Komori et al. 2015; Romme Christensen et al. 2019). The effects of natalizumab and methylprednisolone on these selected biomarkers were previously described (Romme Christensen et al. 2019).

### Disease Activity and Impact

Among clinical outcomes, Expanded Disability Status Scale (EDSS), Multiple Sclerosis Impairment Scale (MSIS), and Combinatorial Weight-Adjusted Disability (CombiWISE) scores were assessed (Kosa et al. 2016; Ratzer et al. 2016; Romme Christensen et al. 2014).

MRI of the brain was performed using a 3 T Siemens Trio scanner (Siemens, Erlangen, Germany) with T1-weighted pre- and post-gadolinium sequences for assessment of contrast-enhancing lesions (Ratzer et al. 2016; Romme Christensen et al. 2014).

We additionally included a combined measure of active PMS at baseline, which was defined as the presence of one or more of the following indicators of inflammatory activity: contrast-enhancing lesions, relapse the previous year, and detectable C-X-C motif chemokine ligand 13 (CXCL13) or matrix metalloproteinase-9 (MMP-9) in CSF by ELISA (Sellebjerg et al. 2017).

### Statistical Analysis

Depending on their distribution, continuous data are presented as mean with standard deviation or median with 25%−75% interquartile values. Categorical data are presented as counts with percentages and compared using Fisher’s exact test. We used paired and unpaired t-tests of log_10_-transformed data to analyze differences in biomarker concentrations. The Mann-Whitney U-test or unpaired t-test was used to analyze differences in clinical characteristics and measures of disease activity between patients with and without IgM OCBs. Proteome data were available for all samples. For ECL, ELISA and imaging analyses, missing data were excluded. Analyses were made in R version 4.2.2 (Core Team 2022; Schauberger and Walker 2023; Wickham et al. 2023) and graphs were made using R (Kolde 2025) and GraphPad Prism version 10.4.1 (La Jolla, USA). For proteome analyses, we used a significance level of *p* < 0.001 with *p* values below this threshold considered statistically significant. Furthermore, FDR-adjusted *q* values were calculated using the Benjamini–Hochberg method. For remaining analyses, we considered *p* < 0.05 statistically significant.

## Results

### Participant Characteristics

Mean age and median progression duration at baseline were 45 ± 8 years and 6 [3;8] years, respectively, for the natalizumab cohort and 49 ± 6 years and 5 [3;9] years, respectively, for the methylprednisolone cohort. The proportion of female participants was 9/17 (53%) in the natalizumab cohort, and 13/23 (57%) in the methylprednisolone cohort. In the natalizumab cohort, 10/17 (59%) were diagnosed with PPMS, and 7/17 (41%) with SPMS, while the distribution was 9/23 (39%) with PPMS and 14/23 (61%) with SPMS in the methylprednisolone cohort. We found no significant difference in the proteome between patients with PPMS and SPMS (Fig. S1).

### Effects of Natalizumab on the Proteome

**Fig. 1:**
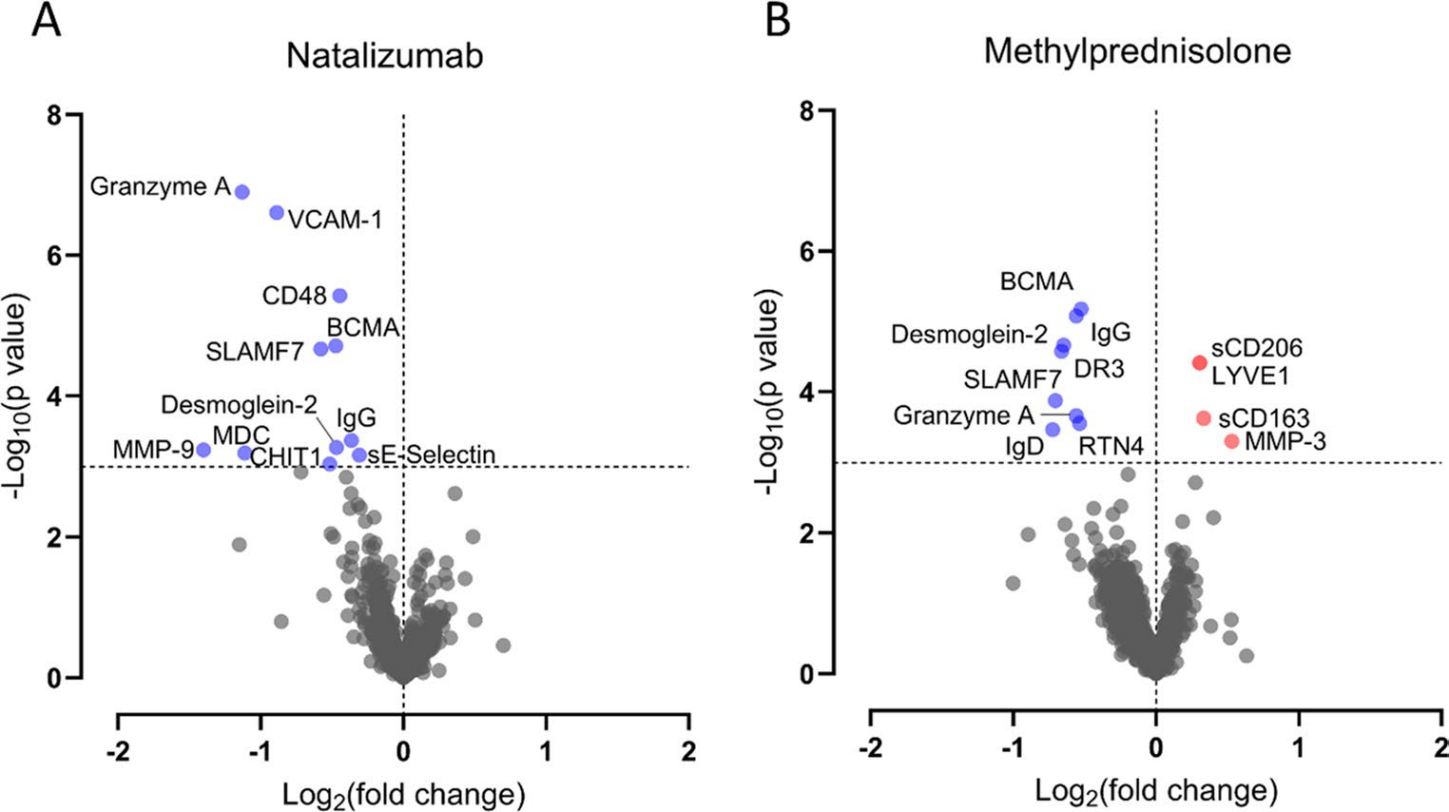
Treatment-associated changes in the cerebrospinal fluid proteome. Volcano plots depicting comparative analyses between untreated baseline CSF samples and week 60 CSF samples. **A**: Changes after natalizumab treatment. **B**: Changes after methylprednisolone treatment. Horizontal dotted lines depict the significance threshold of *p* < 0.001 (paired t-tests of log_10_-transformed concentrations). Vertical dotted lines separate proteins with increased (right) and reduced (left) abundance after treatment

When comparing untreated baseline CSF samples with follow-up samples after 60 weeks of natalizumab treatment, we found significant reductions of 11 proteins. Affected proteins were associated with B cells (B-cell maturation antigen (BCMA), signaling lymphocytic activation molecule family member 7 (SLAMF7), and IgG), T cells (granzyme A), myeloid cells (macrophage-derived chemokine (MDC) and chitinase 1 (CHIT1)), leukocyte activation (CD48 and MMP-9), and cell adhesion (desmoglein-2, vascular cell adhesion molecule 1 (VCAM-1), and soluble E-selectin/endothelial-leukocyte adhesion molecule 1 (sE-selectin)) (Fig. 1A and S2). All treatment effects with *p* < 0.001 also had *q* < 0.1 (Table S1).

### Effects of Methylprednisolone on the Proteome

In analyses of untreated baseline CSF samples and follow-up samples after 60 weeks of methylprednisolone treatment, we found a significant effect on the concentrations of 12 proteins. Like natalizumab, methylprednisolone reduced the concentrations of BCMA, SLAMF7, granzyme A, IgG, and desmoglein-2. Furthermore, there was a reduction of death receptor 3 (DR3), IgD, and reticulon 4 (RTN4), and increased concentrations of lymphatic vessel endothelial hyaluronan receptor 1 (LYVE1), MMP-3, and myeloid cell biomarkers sCD163 and macrophage mannose receptor-1 (sCD206) (Fig. 1B and S2). The affected proteins with *p* < 0.001 also had *q* < 0.1 (Table S1).

### IgM OCBs

**Fig. 2:**
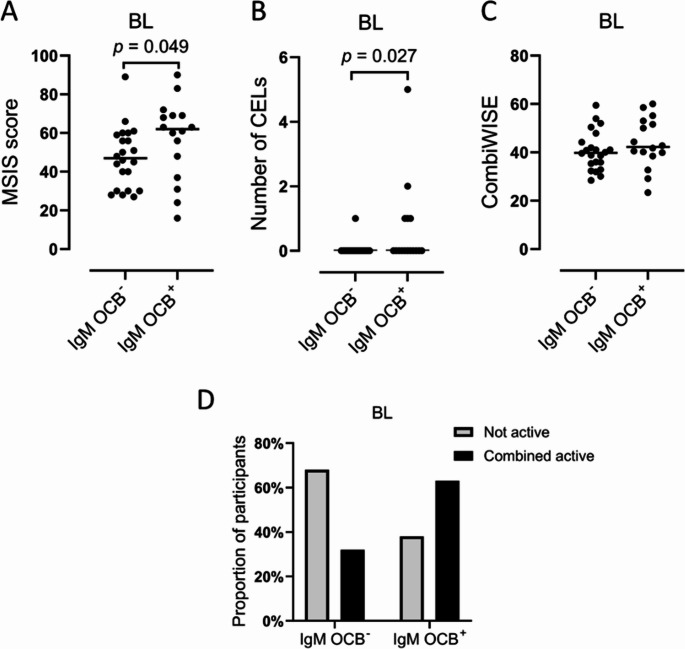
Disease activity and impact according to IgM OCB status.** A**: Multiple Sclerosis Impairment Scale (MSIS) score, **B**: number of contrast-enhancing lesions on MRI scan, **C**: Combinatorial Weight-Adjusted Disability Score (CombiWISE) score, **D**: Proportion of patients with combined active MS. Shown for patients with and without IgM OCBs at baseline. Significant differences are shown with brackets (A-C: Mann–Whitney U tests, D: Fisher’s exact test)

IgM OCBs were detected in 16/38 (42%) unique patients at baseline. In addition, of the two patients who participated in the methylprednisolone trial after the natalizumab trial, one had IgM OCBs at baseline before methylprednisolone, and one did not. At week 60, 2/8 (25%) with IgM OCBs at baseline did not have IgM OCBs after natalizumab treatment, and 3/9 (33%) with IgM OCBs at baseline did not have IgM OCBs after methylprednisolone treatment. All patients without IgM OCBs at baseline (*n* = 23) were still IgM OCB negative at week 60. There were no differences in the MS diagnosis, sex, age, EDSS or progression duration between participants with and without IgM OCBs at baseline (Table S2). Patients with IgM OCBs had a higher median MSIS score and number of contrast-enhancing lesions, while no significant difference was found in CombiWISE score or proportion with combined active PMS (Fig. 2).

**Fig. 3:**
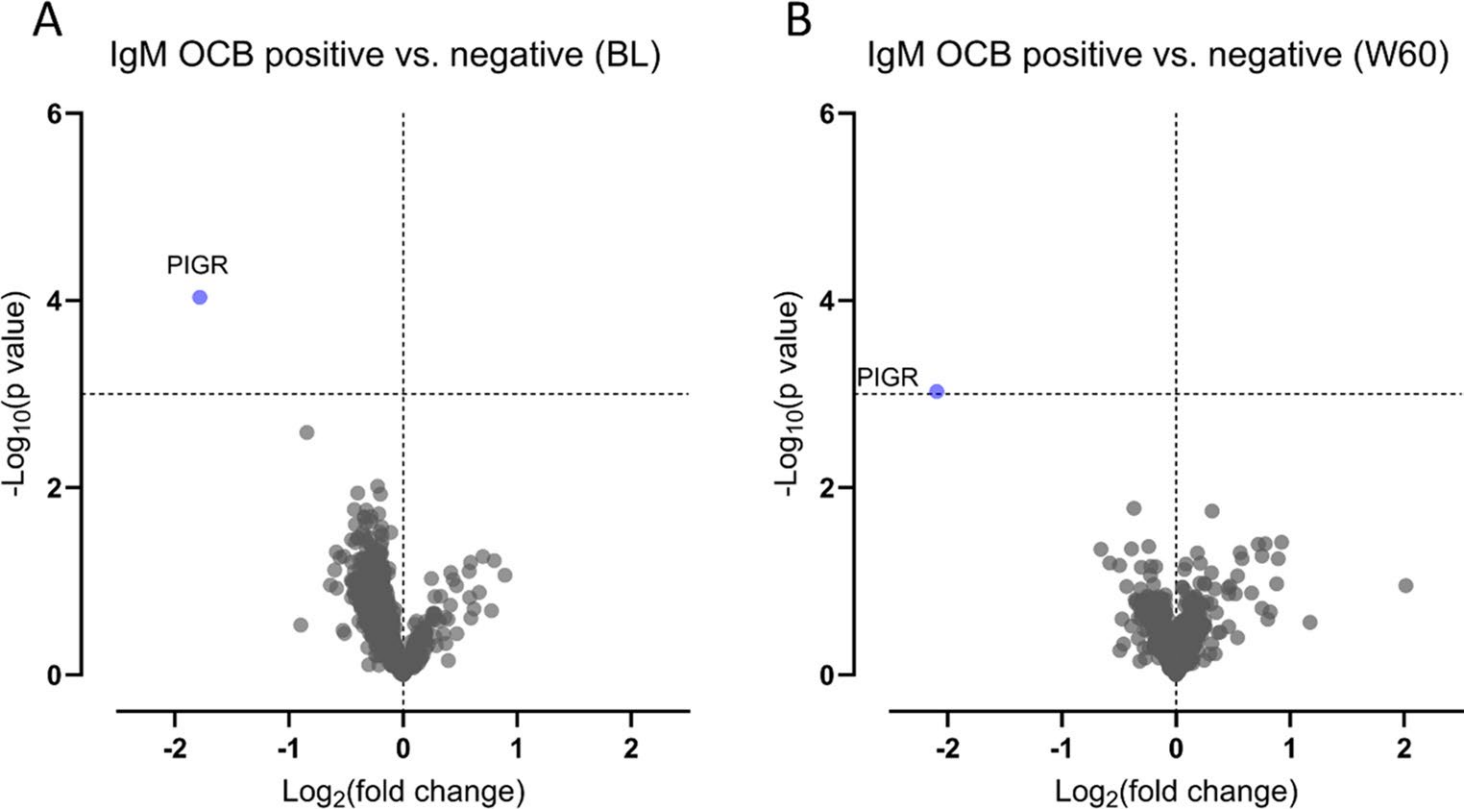
Differences in cerebrospinal fluid proteome according to IgM OCB status. Volcano plots depicting comparative analyses between patients with and without IgM OCBs. **A**: Differences at baseline. **B**: Differences at week 60 after treatment for both cohorts combined. Horizontal dotted lines depict the significance threshold of *p* < 0.001 (unpaired t-tests of log_10_-transformed concentrations). Vertical dotted lines separate proteins with increased (right) and reduced (left) abundance in patients with IgM OCBs

In comparative CSF proteome analyses, we only found a significant difference in the concentration of polymeric immunoglobulin receptor (PIGR) between patients with and without IgM OCBs. IgM OCBs were associated with a lower level of PIGR at baseline (Fig. 3), with *q* = 0.1 at baseline, but *q* = 1.0 at week 60 (Table S3).

**Fig. 4:**
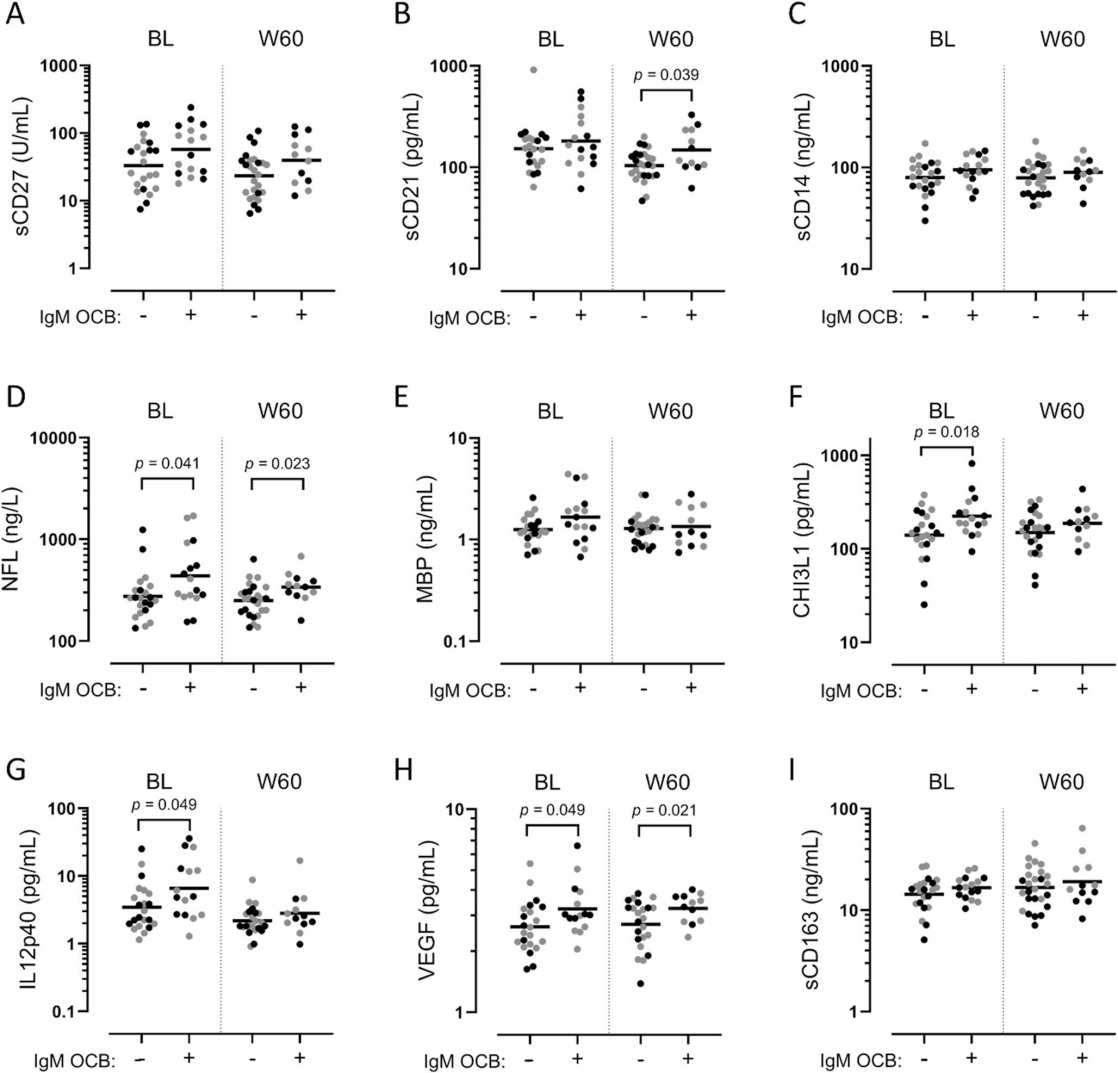
Differences in cerebrospinal fluid soluble biomarker concentrations according to IgM OCB status. Concentrations in patients with and without IgM OCBs of **A**: Soluble CD27 (sCD27), **B**: sCD21, **C**: sCD14, **D**: Neurofilament light chain (NFL), **E**: Myelin basic protein (MBP), **F**: Chitinase-3 like-protein-1 (CHI3L1), **G**: Interleukin-12 subunit p40 (IL12p40), **H**: Vascular endothelial growth factor (VEGF), **I**: sCD163. BL: baseline. W60: week 60. Geometric means are shown. Significant differences are shown with brackets (unpaired t-tests of log_10_-transformed concentrations). Black dots: natalizumab cohort. Grey dots: methylprednisolone cohort

Additional soluble biomarker analyses showed a higher concentration of NFL and VEGF at baseline and week 60, and a higher concentration of CHI3L1 and IL12p40 at baseline in patients with IgM OCBs (Fig. 4). There was no difference in PIGR concentrations in CSF after treatment with natalizumab or methylprednisolone (Fig. S2)

### Fold Change Profiles

To visualize patterns in fold change upon treatment, we generated a heatmap of fold changes in relevant CSF proteins at the participant-level (Fig. S3).

## Discussion

Proteome analysis showed that treatment with natalizumab or monthly methylprednisolone pulse treatment reduced the CSF levels of BCMA, SLAMF7, granzyme A, IgG and desmoglein-2. Additionally, we found natalizumab-specific reductions of adhesion molecules and methylprednisolone-specific increases in myeloid cell biomarkers.

Patients with IgM OCBs had higher levels of CSF NFL and VEGF than patients without IgM OCBs irrespective of treatment status, whereas the level of PIGR was reduced and concentrations of CHI3L1 and IL12p40 higher in untreated baseline samples only. Furthermore, IgM OCBs were associated with a higher MSIS score and contrast-enhancing lesions at baseline.

Interestingly, the affected B cell/plasma cell-related biomarkers BCMA, SLAMF7 and IgG were found to differentiate patients with MS from controls in previous proteomic studies (Åkesson et al. 2023b; Barbour et al. 2017; Held et al. 2025). Whereas the migration of peripheral immune cells to the CNS is considered important for relapse biology, the inflammation in PMS is mainly compartmentalized within the CNS (Hemmer et al. 2015; Komori et al. 2015; Lassmann 2019; Li et al. 2018). Our findings of decreased levels of B cell activity biomarkers after natalizumab therapy may suggest a component of systemic immune cell transmigration in progressive stages of MS as well, in line with the effect of systemic B cell depletion with anti-CD20 monoclonal antibodies in both relapsing and progressive MS (Graf et al. 2021; Hauser et al. 2017; Montalban et al. 2017), and the reductions observed in CSF B cell activity biomarkers (Cross et al. 2024). The findings are also consistent with the reduction in intrathecal IgG synthesis observed in placebo-controlled trials of methylprednisolone treatment in acute optic neuritis and RRMS (Sellebjerg et al. 2000).

The reduction of adhesion molecules by natalizumab therapy is consistent with its mode of action, including a broad downregulation of inflammatory molecules. The interaction between VLA-4 on circulating immune cells and VCAM-1 on endothelial cells facilitates cell extravasation across the blood-brain-barrier (Sellebjerg and Sørensen 2015). Blocking of VLA-4 by natalizumab causes a downregulation of VCAM-1, and previous studies have shown a consequent reduction of the concentration of sVCAM-1 in serum (Millonig et al. 2010; Petersen et al. 2016). Additionally, VLA-4-independent adhesion molecules may be reduced indirectly through a sheddase-mediated mechanism (Millonig et al. 2010).

The increased concentrations of sCD206, sCD163 and LYVE-1 after treatment with methylprednisolone is consistent with the notion that treatment may induce a shift from proinflammatory to homeostatic or immunoregulatory mononuclear phagocyte functions (Mirarchi et al. 2024; Xue et al. 2014).

Notably, previous studies also found CSF concentrations of the treatment-responsive proteins granzyme A, MMP-9, CD48, DR3, desmoglein-2 and RTN4 to be increased in patients with MS compared to controls (Åkesson et al. 2023b; Held et al. 2025; Kulczyńska-Przybik et al. 2021, 2022; Masvekar et al. 2019). While less studied, RTN4 is a candidate biomarker of neurodegeneration (Kulczyńska-Przybik et al. 2021, 2022).

Our findings of more contrast-enhancing lesions and higher concentrations of NFL, CHI3L1, IL12p40 and VEGF in patients with IgM OCBs confirm that the presence of IgM OCBs in patients with PMS identifies a subset with increased focal inflammation (Villar et al. 2014), and support further investigation of whether this subset of patients may benefit more from DMTs (Montobbio et al. 2025; Sellebjerg et al. 2017). Several studies have found a higher NFL elevation in patients with inflammatory disease activity (Sellebjerg et al. 2019; Williams et al. 2021). CHI3L1, IL12p40, and VEGF are mostly secreted by cells from the innate immune system. Though increased levels of CHI3L1 have been associated with disability accrual in PPMS (Pérez-Miralles et al. 2020), CHI3L1, IL12p40 and VEGF have also been associated with acute inflammatory disease activity (Lu et al. 2021; Masvekar et al. 2021; Proescholdt et al. 2002; Su et al. 2006).

The observation of lower CSF PIGR concentrations in people with MS with IgM OCBs is a novel finding. Membrane-bound PIGR is mainly expressed in epithelial cells, where it facilitates transcytosis of IgM and IgA across epithelial layers (Wei and Wang 2021). In its secreted form, PIGR has additional functions, including binding and stabilizing dimeric IgA, inhibiting the chemotactic activity of IL-8 and the complement receptor CR3, and binding to various pathogen molecules (Kaetzel 2005). Although a causal mechanism cannot be inferred from the present study, we hypothesize that the lower concentrations of PIGR in patients with IgM OCBs might reflect higher intrathecal levels of IgM and subsequent binding to membrane-bound PIGR, making less PIGR available for shedding of secretory component. This, in turn, could be associated with less pronounced anti-inflammatory effects of secretory component. Additional studies and further validation in independent cohorts are, however, needed to substantiate this hypothesis.

Important limitations of the study are the exploratory nature of proteomics and that we did not have a validation cohort or orthogonal validation of affected proteins. However, SOMAscan measurements of BCMA have previously been validated using an alternative assay (Barbour et al. 2017), and the reduction of CHIT1 and MMP-9 by natalizumab and increase in sCD163 by methylprednisolone validates our previously published data, where these biomarkers were measured using ECL or ELISA with similar results (Romme Christensen et al. 2014, 2019 and Talbot et al. submitted). In addition, the study is limited by the absence of control groups without treatment at follow-up, as well as controls without inflammatory neurological disease. Furthermore, we had limited power as the original studies were designed and powered to detect treatment effects on osteopontin, and not for the exploratory analyses conducted in this follow-up study (Ratzer et al. 2016; Romme Christensen et al. 2014). Baseline samples may have been impacted by residual treatment effects of previous DMTs, in particular for the methylprednisolone cohort, where fewer patients were treatment-naïve at inclusion, though all participants were untreated for at least 3–6 months. We included both patients with PPMS and SPMS, thus investigating a more heterogenous population in terms of disease course classification. However, this distinction is not reflected in progressive disease biology biomarkers (Barbour et al. 2017; Krieger et al. 2024; Kuhlmann et al. 2023). In line with previous studies, we found no difference in the proteome between patients with PPMS and SPMS. The CSF used for IgM OCBs analysis had undergone one or two thaw-freeze cycles, but considering the relatively high prevalence of IgM OCB positivity in our cohorts, it is unlikely that this has influenced the detection sensitivity. Lastly, CSF findings may not accurately reflect pathophysiological mechanisms within the CNS parenchyma, especially in patients with PMS with more compartmentalized inflammation. The strengths of the study include the systematic longitudinal sampling of CSF using the same protocols, the consistent treatment regimens, and that the patients were untreated at baseline.

In conclusion, the study suggests that BCMA, SLAMF7, granzyme A, IgG and desmoglein-2 are treatment-responsive biomarkers in patients with PMS. Furthermore, we found additional natalizumab-specific reductions of adhesion molecules and methylprednisolone-specific increases of myeloid cell biomarkers which may be associated with dampening of pathogenic mononuclear phagocyte activity. Lastly, we confirm that IgM OCBs are associated with a more inflammatory MRI and CSF profile.

## Supplementary Information

Below is the link to the electronic supplementary material.

Supplementary file 1 (996 KB)

## Data Availability

Anonymized data can be shared upon reasonable request from any qualified investigator. Sharing of data requires approval of a data transfer agreement in accordance with the General Data Protection Regulation and Danish data protection regulation.
