# Supplementary material for: Profiling the Cerebrospinal Fluid Proteome in Progressive Multiple Sclerosis: Treatment Effects and Associations with IgM Oligoclonal Bands
Source: J Neuroimmune Pharmacol. 2025 Oct 30;20(1):98. doi: 10.1007/s11481-025-10263-w (PMC12575587; doi:10.1007/s11481-025-10263-w)
Supplement: Supplementary file 1 — (996 KB) [file 11481_2025_10263_MOESM1_ESM.docx]

**Figure S1**


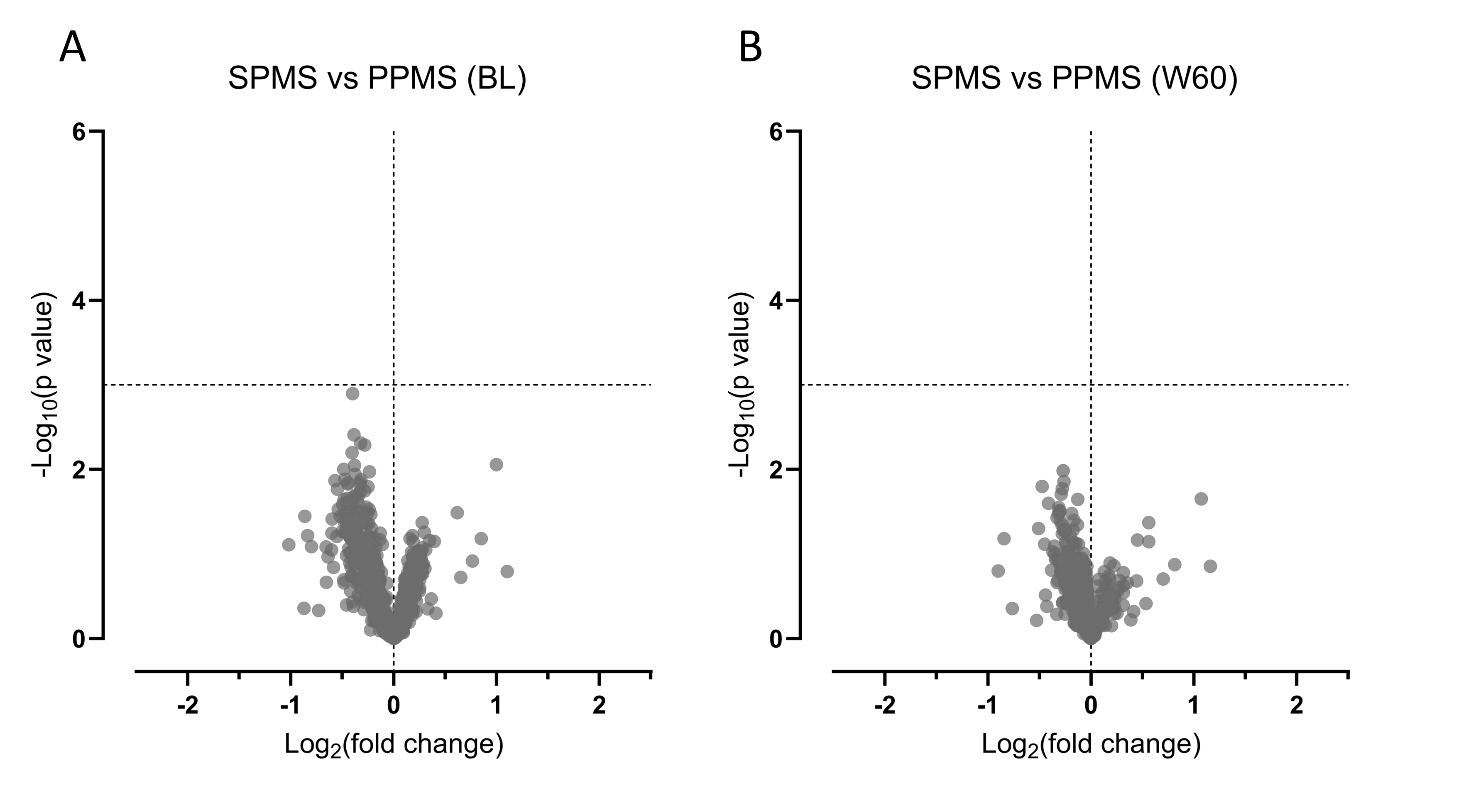


**Differences in the cerebrospinal fluid proteome according disease course.** Volcano plots depicting comparative analyses between patients with PPMS and SPMS. **A**: Differences at baseline. **B**: Differences at week 60 after treatment for both cohorts combined. Horizontal dotted lines depict the significance threshold of *p* <0.001 (unpaired t-tests of log_10_-transformed concentrations). Vertical dotted lines separate proteins with increased (right) and reduced (left) abundance in patients with SPMS.

**Figure S2**


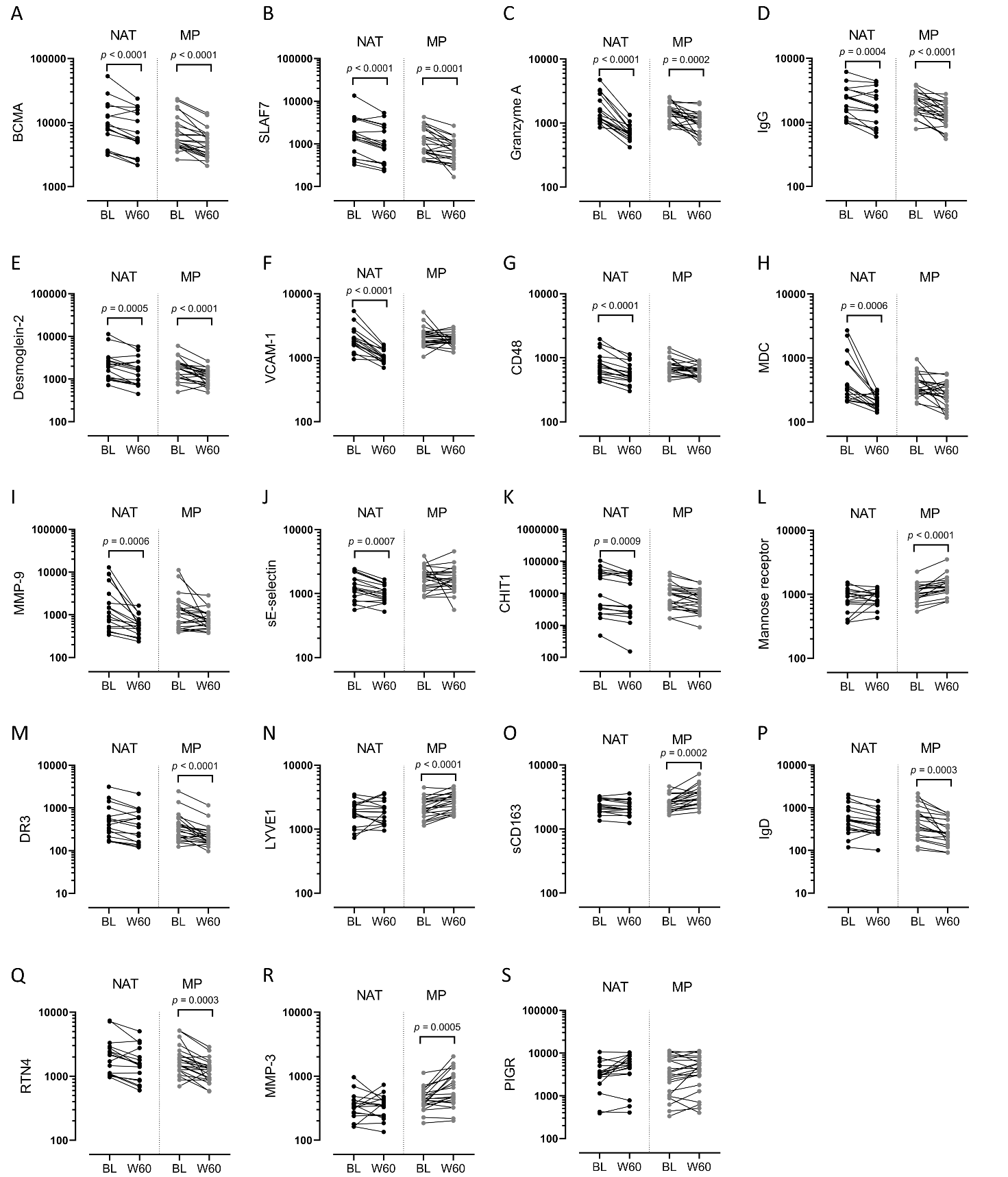


**Individual** **longitudinal changes in cerebrospinal fluid protein concentrations.** A-E: Changes after natalizumab (NAT) and methylprednisolone (MP) treatment. F-K: Changes after NAT treatment only. L-R: Changes after MP treatment only. S: No change was observed in PIGR concentration after treatment. Paired baseline (BL) and week 60 (W60) samples.

**Table S1**

|  | Natalizumab (n = 17) | | | Methylprednisolone (n = 23) | | |
| --- | --- | --- | --- | --- | --- | --- |
| Protein | Fold change | p | q | Fold change | p | q |
| Granzyme A | 0.46 | **<0.0001** | **0.0001** | 0.68 | **0.0002** | **0.0295** |
| VCAM-1 | 0.54 | **<0.0001** | **0.0001** | 0.92 | 0.2850 | 0.4648 |
| CD48 | 0.73 | **<0.0001** | **0.0014** | 0.84 | 0.0042 | 0.3139 |
| BCMA | 0.72 | **<0.0001** | **0.0048** | 0.70 | **<0.0001** | **0.0047** |
| SLAMF7 | 0.67 | **<0.0001** | **0.0048** | 0.61 | **0.0001** | **0.0214** |
| IgG | 0.78 | **0.0004** | **0.0772** | 0.68 | **<0.0001** | **0.0047** |
| Desmoglein-2 | 0.72 | **0.0005** | **0.0772** | 0.64 | **<0.0001** | **0.0073** |
| MMP-9 | 0.38 | **0.0006** | **0.0772** | 0.67 | 0.0205 | 0.4177 |
| MDC | 0.46 | **0.0006** | **0.0772** | 0.73 | 0.0086 | 0.4177 |
| sE-Selectin | 0.81 | **0.0007** | **0.0772** | 0.92 | 0.4377 | 0.5870 |
| Chitotriosidase-1 | 0.70 | **0.0009** | **0.0939** | 0.74 | 0.0045 | 0.3151 |
| DR3 | 0.78 | 0.0024 | 0.1806 | 0.63 | **<0.0001** | **0.0073** |
| sCD206 | 1.06 | 0.5412 | 0.9261 | 1.24 | **<0.0001** | **0.0073** |
| LYVE1 | 1.04 | 0.6527 | 0.9261 | 1.24 | **<0.0001** | **0.0073** |
| sCD163 | 0.95 | 0.0359 | 0.8046 | 1.26 | **0.0002** | **0.0295** |
| RTN4 | 0.76 | 0.0014 | 0.1221 | 0.69 | **0.0003** | **0.0314** |
| IgD | 0.80 | 0.0034 | 0.2413 | 0.61 | **0.0003** | **0.0347** |
| MMP-3 | 0.99 | 0.9074 | 0.9898 | 1.45 | **0.0005** | **0.0467** |
| PIGR | 1.40 | 0.0098 | 0.4897 | 1.17 | 0.1385 | 0.4177 |

Fold changes with p- and q-values for longitudinal changes in cerebrospinal fluid protein concentrations and PIGR.

**Table S2**

**Baseline characteristics depending on IgM OCB status.**

|  | IgM OCB^-^ (n = 22) | IgM OCB^+^ (n = 16) | p |
| --- | --- | --- | --- |
| Age^a^ | 48 (7) | 47 (8) | 0.73 |
| Sex, female, n (%) | 13 (59%) | 7 (44%) | 0.51 |
| Disease duration, years^b^ | 11 [6;18] | 8 [4;14] | 0.47 |
| Progression duration, years^b^ | 7 [4;10] | 6 [3;7] | 0.26 |
| EDSS score^b^ | 4.5 [4.0;5.9] | 4.5 [4.4;6.1] | 0.42 |
| Disease course |  |  |  |
| SPMS, n (%) | 10 (45%) | 10 (63%) | 0.34 |
| PPMS, n (%) | 12 (55%) | 6 (38%) |  |

^a^Mean (SD), ^b^Median [Q1, Q3]

**Table S3**

|  | IgM OCB+ vs. – (BL) | | IgM OCB+ vs. – (W60) | |
| --- | --- | --- | --- | --- |
| Protein | p | q | p | q |
| PIGR | **0.0001** | 0.1039 | **0.0009** | 0.9968 |

P- and q-values for proteomic differences between participants with and without IgM OCBs at baseline and week 60.

**Figure S3**


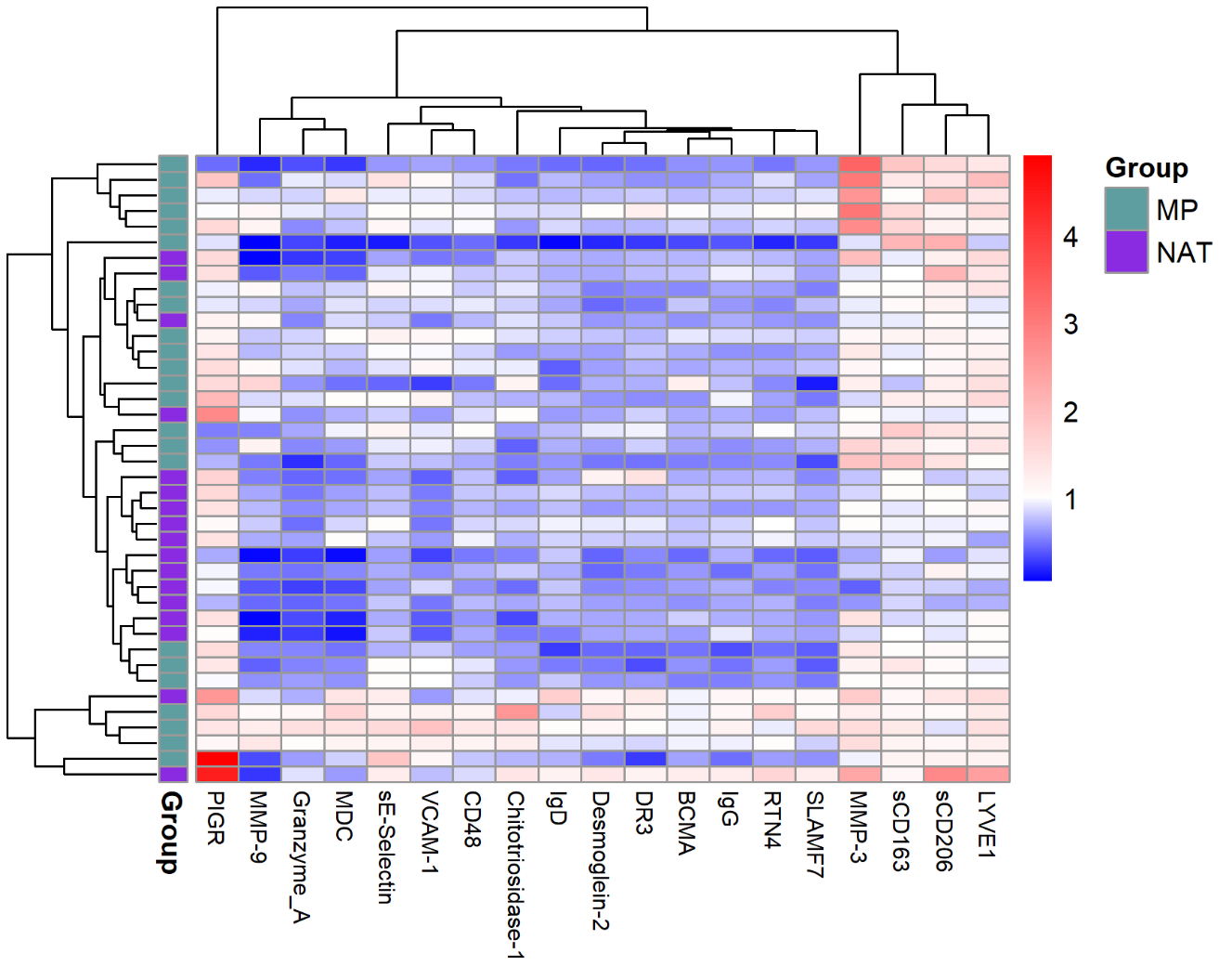


**Fold change patterns**. Clustered heatmap showing the fold change in relevant cerebrospinal fluid proteins after methylprednisolone (MP) or natalizumab (NAT) treatment. Only proteins affected by treatment or IgM OCB status were included. Each row represents one participant.
